# Comparison of Minimally Invasive Fixation of Mid‐Shaft Clavicular Fractures with Threaded Elastic Intramedullary Nail (TEIN) and Elastic Locking Intramedullary Nail (ELIN)

**DOI:** 10.1111/os.13129

**Published:** 2021-10-04

**Authors:** Liang Ren, Zhiqiang Yang, Yongqing Wang, Sapkota Basanta, Bosong Du, Zhuo Gao, Baoxi Hao, Renhui Chen

**Affiliations:** ^1^ School of Chinese Integrated Chinese and Western Medicines Tianjin University of Traditional Chinese Medicine Tianjin China; ^2^ Orthopaedics Department The Fourth Central Hospital Affiliated to Nankai University Tianjin China; ^3^ Huazhong University of Science and Technology Tianjin China

**Keywords:** Clavicle, Elastic intramedullary nail, Faster osseous healing, Fracture, Intramedullary fixation

## Abstract

**Objective:**

To compare the effectiveness of threaded elastic intramedullary nail and elastic locking intradullary nail (ELIN) for mid‐shaft clavicular fractures.

**Methods:**

The clinical data of 47 patients with middle clavicle fracture treated by TEIN and ELIN from August 2017 to March 2019 were analyzed retrospectively. Twenty‐three patients received intramedullary fixation treatment with ELIN, nine males and 14 females, AO/OTA fracture classification type 2A (n = 17) and 2B (n = 6). Twenty‐four patients received intramedullary fixation treatment with TEIN, including nine males and 15 females, AO/OTA classification: type 2A (n = 18) and 2B (n = 6). All patients were anesthetized with ipsilateral cervical plexus block. After internal fixation was removed, the clinical outcomes were assessed and evaluated. The Constant‐Murley score and disabilities of the arm, shoulder and hand questionnaire (DASH) score were compared between the two groups to evaluate the functional status of all patients. The study was done accordingly to the guidelines provided by the ethics committee.

**Results:**

All patients in the two groups completed the operation successfully and were followed up. In the ELIN group, the operation time was 20.78 ± 7.71 min, intra‐operative blood loss was 13.26 ± 9.72 mL, incision length was 1.60 ± 0.92 cm, internal fixation removal time was 12.86 ± 2.24 weeks, Constant‐Murley score was 99.30 ± 1.36 points and DASH score was 1.43 ± 3.00 points. In the TEIN group, the operation time, intra‐operative blood loss, incision length, internal fixation removal time, Constant‐Murley and DASH scores were 22.83 ± 8.17 min, 22.08 ± 11.22 mL, 2.48 ± 0.84 cm, 15.66 ± 5.58 weeks, 95.79 ± 7.38 point and 6.69 ± 11.55 point respectively. In the ELIN group, four cases developed skin irritation, and the symptoms were relieved after removal of internal fixation. In the TEIN group, one patient's internal fixation broke and had an obvious scar at the incision, but there was no fracture after replacement of internal fixation; withdrawal of TEIN occurred in four patients, the nail did not shift again until the last follow‐up; skin irritation and temporary bursitis occurred in six patients, and the symptoms were relieved after internal fixation was removed. No other conditions were found in the patients, and bony healing was achieved in all patients.

**Conclusion:**

ELIN prevents shortening and malunion of the clavicle, reduces secondary damage to related tissues, and leads to restoration of clavicle length and faster osseous healing.

## Graphical abstract

Flowchart of Tein and Elin in the treatment of mid‐clavicle fracture

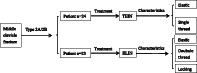

## Introduction

The clavicle is the connection and support device between the upper limb and the torso, which is S‐shaped. The common mechanism of clavicle injury is to reach out and fall and injure oneself. Clavicle fractures are common in adults and children, most frequently occurring in persons who are younger than 25 years old. Clavicle fractures account for 2.6% of all fractures, of which more than 80% of clavicle fractures involve the middle^1^, where the typical compacting forces results in bony failure which is applied to the shoulder and the narrow cross section of the bone^2^. In recent years, the incidence of clavicle fracture is on the rise. Clinical treatment can be divided into conservative treatment and surgical treatment. Some studies suggest that conservative treatment has good results for bone healing and shoulder function recovery^3^, ^4^. However the various disadvantages of conservative treatment occur such as instability^5^, phlebostasis of arm veins^6^, displacement of fracture end and high nonunion rate^7^, ^8^, axillary decubitus ulcers and compression of the neurovascular bundle^9^. Due to poor results, surgical treatment for displaced middle clavicle fracture has become a widespread consensus^10^. There are two types of surgical treatment for displaced clavicle fracture. One is open reduction plate internal fixation^6^, ^11^, ^12^, ^13^, ^14^ including ordinary plate, AO plate and anatomical plate internal fixation. However, there are extensive peeling of periosteum, tissue injury and removal of local hematoma in plate internal fixation, which destroy the regeneration environment of the fracture site and the source of osteocyte growth, which seriously affect the rehabilitation of fractures. Plate fixation causes local stress shielding, interferes with stress stimulation, affects fracture healing, and surgical incisions form large scars, affecting beauty and so on. Another surgical method is intramedullary fixation, like Rockwood pins^8^, Knowles pins^15^, Hagie pins^16^, which have their own merits and demerits.

Based on the problems of plate and intramedullary fixation, and the anatomical characteristics of clavicular marrow filled with cancellous bone, we designed TEIN, which The sternal end of Tein is a cone, and the surface is higher than the thread of the nail body, which is locked with the cancellous bone of the medullary cavity. It has a good result for the treatment of clavicle fracture with closed reduction and intramedullary fixation. However, because TEIN only designs the thread at the head end to lock with the proximal clavicle, it merely has the effect of preventing nail body movement, but the anti‐shortening function is not ideal, so some patients have clavicle shortening deformity and acromial skin irritation, even penetrate the skin to form local bedsores. In order to solve the problems such as poor anti‐shortening ability of TEIN and abnormal healing of clavicle shortening after operation, ELIN was designed on the basis of TEIN to treat middle clavicle fracture. The data of minimally invasive treatment of middle clavicle fracture with ELIN in Tianjin fourth Central Hospital from August 2017 to March 2019 was retrospectively analyzed and compared with TEIN treatment of middle clavicle fracture. The main aims of this study were: (i) to compare the differences in operative time, intraoperative incision length, blood loss, fracture healing, and functional recovery between the two types of elastic intramedullary nails; (ii) to explore the mechanism of rapid recovery of fracture accelerated by Elastic Osteosynthesis (EO) theory in elastic fracture fixation; and (iii) to summarize the experience of minimally invasive treatment of middle clavicle fracture.

## Materials and Methods

### Inclusion Criteria

Inclusion criteria for this study were: (i) clavicle fracture, AO/OTA classification is type 2A and 2B; (ii) patients treated with ELIN; (iii) patients treated with TEIN were used as control; (iv) the main evaluation indicators were clinical data, postoperative shoulder joint and upper limb function; and (v) retrospective case analysis.

### Exclusion Criteria

Exclusion criteria for this study were: (i) open fracture; (ii) pathological fracture; (iii) combined with injury of peripheral nerve and blood vessels of shoulder joint, or previous history of shoulder surgery; (iv) Age ≤ 10 years old; and (v) the follow‐up period is less than 3 months.

### Patients

From August 2017 to March 2019, middle clavicle fractures were treated in Tianjin fourth Central Hospital. According to the inclusion and exclusion criteria, a total of 47 patients were enrolled in this study. Twenty‐three cases were treated with ELIN fixation, nine males and 14 females. The mean age of the patients was 54.48 years. The clavicle fractures were classified according to the AO Foundation and Orthopedic Trauma Association (AO/OTA) fracture classification compendium^17^, type 2 is diaphyseal fracture of clavicle. A is simple fracture and B is wedge fracture. 17 were type 2A, and 6 were type 2B. Twenty‐four cases were treated with TEIN fixation, nine males and 15 females. The mean age of the patients was 43.08 years. The clavicle fractures were classified according to the AO/OTA classification fracture classification compendium, 18 were type 2A, and six were type 2B. Two groups of patients were given the symptomatic treatment and examination in department. And the operation was performed for middle clavicle fracture on the average of 3.52 ± 1.34 days in ELIN and 3.41 ± 0.88 days in TEIN (Table 1).

**TABLE 1:** General information of patients in the two groups

Group	Age (years)	Gender		AO/OTA classification		Time from trauma to surgery (days)
		Male	Female	Type A	Type B	
ELIN	54.48 ± 16.56	9	14	17	6	3.52 ± 1.34
TEIN	43.08 ± 15.90	9	15	18	6	3.41 ± 0.88
*Statistic*	*t* = 2.406	*X^2^* = 1.079		*X^2^* = 1.319		*t* = 0.318
*P value*	0.866	0.758		0.517		0.752

### Materials

**Fig. 1:**
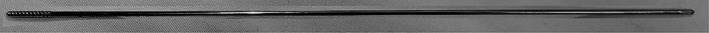
Physical diagram of thread elastic intramedullary nail. The diameters of the TEIN can be divided into 1.5 mm and 2.0 mm, with a length of 260 mm. TEIN is made of stainless steel (317 L). The nail head end is a positive cone, followed by a thread with 15–20 mm length and height higher than the 1.0 mm of the nail body, and the nail body has a certain elasticity.

**Fig. 2:**
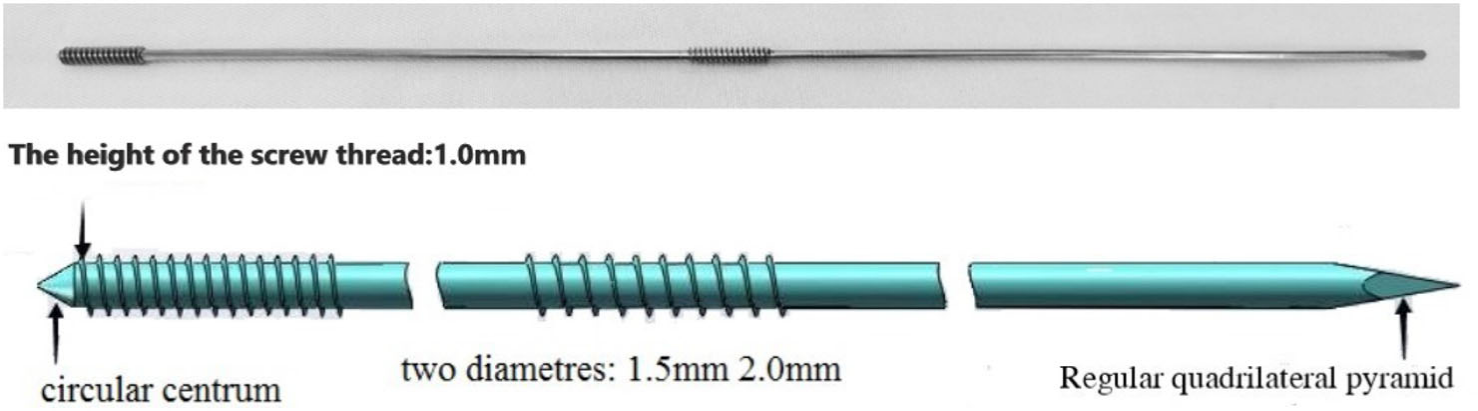
Physical and schematic diagram of the Elastic locking intramedullary nail. ELIN is available in 1.5 mm and 2.0 mm diameters, with a length of 260 mm. ELIN is made of stainless steel (317 L). The nail head end is a circular centrum, followed by a thread with 15–20 mm length and height higher than the 1.0 mm of the nail body, and the nail body has a certain elasticity. ELIN adopts a double thread locking design, and a long 15–20 mm thread higher than the 1.0 mm of the nail body is added at the distance from the head thread 80‐100 mm, which is used to lock the distal end of the fracture. The nail tail end is a regular quadrilateral pyramid, which can pierce the skin easily.

According to the morphological characteristics of clavicle, TEIN (Fig. 1) and ELIN (Fig. 2) were designed. The diameters of the two kinds of elastic intramedullary nails can be divided into 1.5 mm and 2.0 mm, with a length of 260 mm. Both of them are made of stainless steel (317 L). The nail head end is a positive cone, followed by a thread with 15–20 mm length and height higher than the 1.0 mm of the nail body, and the nail body has a certain elasticity. The difference is that the ELIN adopts a double thread locking design, and a long 15–20 mm thread higher than the 1.0 mm of the nail body is added at the distance from the head thread 80‐100mm, which is used to lock the distal end of the fracture. The two kinds of elastic intramedullary nails are made by Kang Li Min Medical Devices, Tianjin, China, and production license and clinical use license have been obtained.

### Operative Procedure

#### Anesthesia and Posture

Both groups were anesthetized with ipsilateral cervical plexus block and placed on the orthopedic operation bed for fluoroscopic. The patient was in supine position and placed a pillar‐shaped pillow on the medial edge of the scapula to make an angle of 30° between the patient's back and the operating table to facilitate shoulder abduction and to promote fracture reduction. The shape of the clavicle was marked and the location of the fracture was determined.

#### Operation of ELIN Fixation

**Fig. 3:**
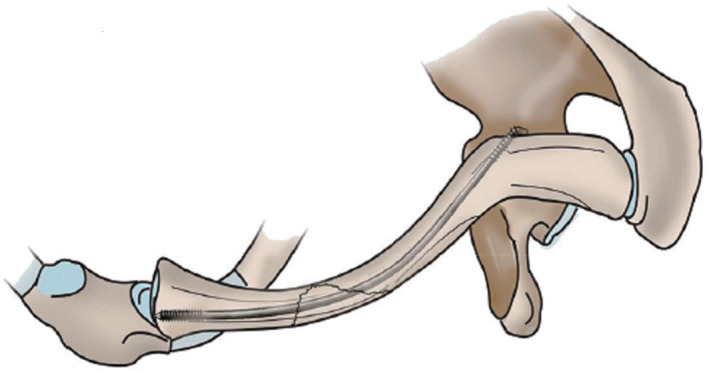
Schematic diagram of clavicle fracture fixed with ELIN. The head thread of ELIN can fix the cortical bone of the proximal clavicle and the cancellous bone of the medullary cavity. The middle thread of ELIN is fixed with the cortical bone of the distal end of the fracture to achieve the function of locking both ends of the fracture. At the end of the operation, the doctor bent and cut the ELIN, and buried the nail tail into the skin.

Two percutaneous reduction clamps were used to slide down against the surface of the clavicle, holding the medial and lateral ends of the clavicle. Primarily, the lateral end was elevated then the distal medullary cavity was inserted by using an awl. According to the preoperative measurement of the size of the clavicular medullary cavity, the elastic intramedullary nail with a diameter of 1.5 mm or 2.0 mm was selected, the head end of elastic intramedullary nail was attached to the drill and drilled carefully under C‐arm radiographic control until it came out at the acromial end of clavicular tubercle. Due to the pyramidal nodules on the lateral side of the clavicle lead to a flattening and narrowing of the clavicular myeloid cavity, the phenomenon of the stagnation of intramedullary nail often occurs, which lead to nail breakage. If necessary, the equivalent Kirschner needle could be used for pre‐expansion. Second, the medial bone reduction clamps were used to elevate the broken end of the medial fracture to keep both ends of the fracture at the same level, and then the shoulder was abducted for reduction of the fracture. Then the intramedullary nail was retrograde inserted into the medullary cavity of the proximal fracture to the sternum of the clavicle and fixed with the cortical bone of the proximal clavicle and the cancellous bone of the medullary cavity. The middle thread of ELIN is fixed with the cortical bone of the distal end of the fracture to achieve the function of locking both ends of the fracture. Two bone reduction clamps were removed at the same time. Flexion and abduction of the shoulder joint to confirm that the internal fixation is stable. At the end of the operation, the doctor bent and cut the ELIN, and buried the nail tail into the skin. Reduction of the fracture and stabilization of elastic intramedullary nail was securted by C‐arm. If closed reduction failed, then limited open reduction and internal fixation were used. Drainage strips were placed in the incision, sutured and covered with aseptic dressing (Fig. 3).

#### Operation of TEIN Fixation

The operation process of TEIN is basically the same as that of ELIN. TEIN has only one threaded at the proximal end, and can only be fixed with the cortical bone of the proximal clavicle and the cancellous bone of the medullary cavity. At the end of the operation, the doctor bent and cut the TEIN, and buried the nail tail into the skin like the ELIN.

### Postoperative Rehabilitation

None of the patients were given antibiotics after operation. A shoulder sling was applied to all the patients aiming to support the affected limb. All the postoperative patients were asked to do exercises of handshake, wrist and elbow joint immediately. Active and passive flexion, extension and abduction painless exercise of shoulder joint were performed on the third day after operation. The patients of closed reduction and internal fixation could be discharged the next day. The patient's with minimally invasive limited open reduction and internal fixation drain was removed 1 day after operation, the wound should be dressed every 3 days, the stitches removed 9 days after operation.

### Outcome Measures

#### Clinical Evaluation

The operation time, intraoperative blood loss and incision length were compared between the two groups.

#### Fracture Healing Evaluation

The patients were tested taking by an anteroposterior (AP) X‐ray examination of the clavicle at the 2nd, 4th, 8th, 12th and 16th weeks after operation to evaluate the effect of fracture healing. In addition to the X‐ray indication that the continuous callus passed through the fracture line and the fracture line was blurred, the callus density at the broken end of the fracture was consistent with the clavicular cortical bone as the real fracture healing, which was not only the most important standard of fracture healing, but also a standard for the removal of internal fixation. Taking the bilateral clavicle AP X‐ray examination, the shape of the bilateral clavicle was compared to evaluate the therapeutic effect of clavicle fracture.

#### DASH and Constant‐Murley Score

The Constant‐Murley score^18^ and disabilities of the arm, shoulder and hand questionnaire (DASH) score^19^ were used. The aim of the Constant‐Murley score was to evaluate the pain, activities of daily living, and strength and range of motion of the shoulder joint. The ratio was 35/65 points, and the full score was 100 points. The function of the shoulder joint was proportional to the score. The DASH score ranges from 0 (disability) to 100 (severe disability) was evaluated the level of disability of the shoulder and arm.

### Statistical Analysis

In this retrospective case study, all the analyses were completed using the SPSS 17.0 Windows (SPSS Inc., Chicago, IL, USA) statistics program. Age, time from trauma to surgery, operation time, incision, intraoperative bleeding, fixation removal time, Constant‐Murley and DASH score were analyzed using T‐test and presented as the median and standard deviations. Gender, classification, and fracture healing were analyzed using Chi‐square test. Significance was set at *P* < 0.05.

## Results

### General Results

No patient was lost to the follow‐up. All the operations were done from 12 to 44 min; the operation time of the ELIN group was 20.78 ± 7.71 min, the TEIN group was 22.83 ± 8.17 min. Blood loss of the ELIN group was 13.26 ± 9.72 mL, the TEIN group was 22.08 ± 11.22 mL. The length of incision of the ELIN group was 1.60 ± 0.92 cm, that was shorter than the TEIN group (2.48 ± 0.84 cm). Except for the time of operation, the differences of other indexes were statistically significant (Table 2).

**TABLE 2:** Time from trauma to surgery, operation time, incision length and Intraoperative bleeding in the two groups

Group	Operation time (min)	Incision (cm)	Intraoperative bleeding (mL)
ELIN	20.78 ± 7.71	1.60 ± 0.92	13.26 ± 9.72
TEIN	22.83 ± 8.17	2.48 ± 0.84	22.08 ± 11.22
*t value*	1.020	3.411	4.059
*P value*	0.381	0.001	0.000

### Intraoperative Observation

**Fig. 4:**
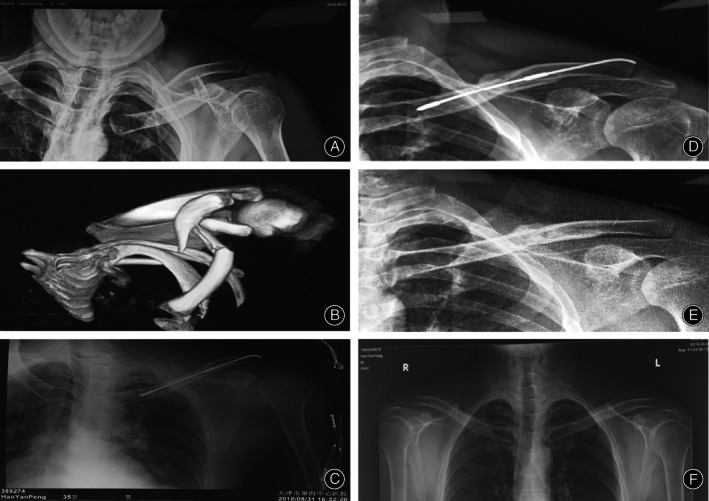
Patient name: Hao Yanpeng. Gender: male. Age:35 years. Injury mechanism: the patient accidentally fell and landed on his left shoulder while riding a bicycle. The patient acute pain and limited movement, and go to the hospital examination immediately. Hospital examination: The examination revealed obvious local swelling and deformity at the left clavicle, obvious tenderness and abnormal movement at the touch of the clavicle. (A) Preoperative X‐ray showing a patient with mid‐shaft clavicular fracture. The distal part of the clavicle shifts upward and backward, and the proximal part of the clavicle shifts downward and forward. (B) Preoperative Three‐dimensional reconstruction image showing AO/OTA classification of type 2B comminuted fracture of middle clavicle, it is a butterfly fracture. (C) Postoperative X‐ray showing good alignment of fracture, distal and proximal ELIN thread and cancellous bone locking nail. (D) One month after operation, X‐ray showing the end of the fracture was in good alignment, there was no loosening of internal fixation, and the intermittent callus passed through the end of the fracture. (E) The X‐ray before and after internal fixation 14 weeks after operation showed that the line of clavicular fracture was blurred and the density of callus was consistent with that of clavicular cortex. (F) Seven months after operation, the comparison between the affected side of the clavicle and the healthy side of the clavicle was basically the same.

The operations on all 47 patients were successful. In the ELIN (n = 8) and TEIN groups (n = 7), the elastic interlocking intramedullary nail was inserted directly through the acromion after closed reduction. In other patients, the intramedullary nail inserted under direct vision because the elastic interlocking intramedullary nail could not enter the clavicular medullary cavity or the reduction was not satisfactory. In all patients who were treated with open reduction and internal fixation, the intraoperative fracture classification was consistent with the preoperative imaging judgment. Postoperative fluoroscopy of the affected shoulder joint showed that the shape of the clavicle returned to normal, and the position of the intramedullary nail was good (Fig. 4).

### Fracture Healing

The implant was removed of ELIN and TEIN group at 12.86 ± 2.24 and 15.66 ± 5.58 weeks respectively, the time of the ELIN group was shorter than that of the TEIN group. All patients was taken the bilateral clavicle AP X‐ray examination to observe the shape of the clavicle on both sides. There was no malunion and shortening of the clavicle in the ELIN group, while there were four cases of shortening and malunion of the clavicle in the TEIN, and the normal healing rate was 83.33% (Table 3).

**TABLE 3:** Therapeutic effect of two groups

Group	Fixation removal time (weeks)	Fracture healing (cases/%)	Constant‐Murley socre	DASH socre
ELIN	12.86 ± 2.24	23/100	99.30 ± 1.36	1.43 ± 3.00
TEIN	15.66 ± 5.58	20/83.33	95.79 ± 7.38	6.69 ± 11.55
*Statistic*	t = 3.253	X^2^ = 2.768	t = 2.891	t = 2.768
*P* value	0.002	0.007	0.005	0.007

### Constant‐Murley and DASH Score

No patients complained of functional limitation of the shoulder joint in the two groups. Constant‐Murley scores in ELIN and TEIN groups were excellent in (n = 22, 18), good (n = 1, 5), fair (n = 0, 1), and poor (n = 0, 0) patients respectively. All patients in the ELIN group achieved excellent strength, less pain, and satisfactory daily life activities with good range of motion, that were better than the TEIN group

The DASH score in the ELIN group was excellent in (n = 35) and good in (n = 3). The DASH score was 1.43 ± 3.00, which was lower than that of the TEIN group (6.69 ± 11.55). No disability of the shoulder and arm was observed in the two groups (Table 3).

### Complications

No patients suffered from infection, neuromuscular compromise, shortening, re‐fracture or nonunion in both groups, and bony healing was achieved. Four patients in the ELIN group suffered from skin irritation post‐operation, and the symptoms were relieved when internal fixation removed. No migration of the ELIN occurred. One patient in the TEIN group had a breakage of the nail and an obvious scar, but there was no breakages when replacing the nail. Withdrawal of TEIN occurred in four patients, and the forearm was given suspension, the nail did not shift again until the last follow‐up. Skin irritation and temporary bursitis occurred in six patients, and the symptoms were relieved after internal fixation was removed. No revision surgery was required for any patient in both groups.

## Discussion

The clavicle is S‐shaped tubular bone, which is flat, tubular and funnel‐shaped from outside to inside, respectively. The junction of the middle and outer 1/3 of the clavicle is the narrowest part of the bone marrow cavity, and it is also the site of high incidence of fracture^20^, ^21^. The anatomical characteristics of the clavicle determine that the two kinds of intramedullary nail bodies must be elastic to adapt to the shape of the interlocking bone marrow cavity to achieve complete elastic intramedullary fixation, so that the stress can be dispersed and the nail breakage can be effectively prevented. That is the basic principle of ELIN is EO (elastic osteosynthesis). ELIN can ensure that it passes through the narrowest part of the clavicle medullary cavity without reaming. The caudal end of ELIN is a flat triangular or triangular pyramidal blade, which can successfully penetrate the lateral cortical bone of the conical tubercle. ELIN adds a double thread locking function on the basis of the advantages of TEIN. The proximal thread lock can rotate along the inner wall of the clavicle and is not easy to pierce the cortical bone. It locks the broken end of the fracture together with the middle thread lock of ELIN, which can resist shortening and maintain the suspension stability and reduction state of the broken end of the fracture. It can avoid complications such as nail migration, clavicle shortening and malunion, affecting clavicle length leading to shoulder asymmetry, beauty and shoulder function^22^. At the same time, double thread locking can provide continuous strength at the broken end of the fracture and stimulates callus growth and promotes fracture healing rapidly. Therefore, the fracture healing and internal fixation were removed earlier in the ELIN group, no malunion and shortening occurred, the functional score was better than that in the TEIN group, and the incidence of skin irritation, pressure sores and other complications in the ELIN group was significantly lower than that in the TEIN group.

Wolff's law^23^ states that healthy bone will adapt to the loads it is placed under. At present, it is considered that the application of longitudinal stress stimulation at the fracture end can accelerate the fracture healing process and shorten the fracture healing time^24^. Rotation, bending and shear stress is not conducive to fracture healing. According to our clinical research and evidence‐based data, we found that the broken end of fracture is subjected to intermittent longitudinal, rotational, bending and shear stress, which can promote fracture healing^25^, ^26^.

Plate fixation can provide strong fixation in the early stages, but the plate fixation is too strong, which eliminates the micro‐stimulation caused by moderate fretting at the broken end, and the stress shielding effect is serious, which affects bone healing^27^. Therefore, the fracture healing delays under the plate fixation, and the re‐fracture will occur after the internal fixation is removed^28^. In this study, ELIN can fix both ends of the fracture to eliminate the axial continuous compressive stress at rest, that is, anti‐shortening locking. The rebound force of ELIN can produce elastic strain with the change of stress, resulting in elastic fixation and small stress shielding. When the tension of muscle, ligament and other tissues is balanced with the elastic deformation of intramedullary nail, the clavicle fracture is in the state of reduction, and the stress shielding of the nail is zero. The broken end of fracture can accept intermittent appropriate stress stimulation to promote fracture healing during painless exercise. From the treatment results, the speed of fracture healing has been greatly improved, indicating that the intermittent appropriate rotation, bending and shear stress has no adverse effect on clavicle fracture healing, but the driving force to promote rapid fracture healing. Previous studies have found that fracture reduction and internal fixation can cause varying degrees of injury to bone, periosteum, muscle, blood vessels and nerves, skin and subcutaneous tissue, and destroy the environment of fracture healing. Among them, the destruction of blood circulation and tissue inflammatory edema are important factors of poor anti‐infection and decreased ability of fracture healing. In the past, there was a lack of understanding of the original hematoma of fracture and was often purposely removed. However, original hematoma removal has a greater impact on fracture healing. Some studies have found that removal of original hematoma can cause delayed union or nonunion of fractures. Because the hemorrhage comes mainly from the bone marrow cavity, the serum levels of alkaline phosphatase, osteocalcin and carboxyl terminal peptide of type I procollagen are significantly increased, which promote the proliferation of osteoblasts and related growth factors and promote the differentiation of mesenchymal cells and periosteal formation of periosteal cells^29^. Therefore, hematocele is the main body of callus formation at the broken end of the fracture, and the removal of hematoma is equal to the elimination of “seed” cells. When the surgery requires open reduction, we removed the local hematoma completely and preserved it on sterile gauze and refilled it to the fracture end when we were done with the reduction. How to reduce reinjury is a key problem in the treatment of fracture.

Clavicular fracture fixation with ELIN is a minimally invasive operation. It is a new direction in the development of Orthopedics^30^. Minimally invasive reduction and intramedullary elastic locking fixation are realized with minimal trauma and the simplest operation in the treatment of middle clavicle fracture with ELIN which is suitable for the most clavicle fracture. Compared with TEIN, ELIN can create continuous stress stimulation at the fracture site and keep the fracture site relatively stable, and prevent shortening deformation and malunion, reduce the secondary damage to related tissues, and obtain the best treatment effect with the most reliable fixation, that is, minimally invasive, beautiful and accelerate bone healing. So the surgeons recommend ELIN for the middle clavicle fracture.

This study has a small number of patients, and ELIN is prohibited for type C clavicle fracture. Also, this is a single center study, a randomized controlled study with large population is needed in multiple centers to further assess the clinical outcomes of ELIN. And the use of ELIN in other tubular fractures requires further study, as well as changes in the diameter and length of ELIN.

## Conflict of Interest Statement

We declare that we have no financial and personal relationships with other people or organizations that can inappropriately influence our work, there is no professional or other personal interest of any nature or kind in any product, service and/or company that could be construed as influencing the position presented in, or the review of the article. Each author certifies that he or she has no commercial associations (e.g., consultancies, stock ownership, equity interest, patent/licensing arrangements, etc.) that might pose a conflict of interest in connection with the submitted article.
